# Manufactured meals: The challenges of ultraprocessed foods

**DOI:** 10.1371/journal.pmed.1004477

**Published:** 2024-10-15

**Authors:** Alexandra Tosun, on behalf of the PLOS Medicine Editors

**Affiliations:** Public Library of Science, San Francisco, California, United States of America, and Berlin, Germany

## Abstract

In this Editorial on behalf of the PLOS Medicine Editors, Alexandra Tosun discusses how ultra-processed food has found itself at the center of a growing storm of criticism, the complexities of the ongoing nutrition debate and why stakeholders must be held to higher standards.

“If you are what you eat, then I only want to eat the good stuff.” Remy, the food-loving rat in the Pixar movie Ratatouille, clearly understood the importance of eating healthy, high-quality food. Yet, focusing on healthy eating is far from straightforward for the average consumer, particularly with the ever-increasing lineup of culinary bad guys on the world’s dinner plates. Ultraprocessed foods (UPFs) have found themselves at the center of a growing storm of criticism, with the mainstream media regularly sounding alarm bells about increased health risks for those whose diets are dominated by these foods.

And consumers might be right to worry. According to a recent umbrella review, there is “convincing” (but mainly low-quality) evidence for a direct association between high UPF consumption and certain adverse health outcomes, including type 2 diabetes, cancer, and even death [1]. But understanding just how risky it is to stroll down the junk food aisle is far from simple. The public is forced to balance competing messaging about UPFs from the mainstream media, which tends to oversimplify and sensationalize the findings of complex and challenging nutrition studies, and a powerful and influential food industry that touts taste, convenience, and affordability.

One source of uncertainty for consumers is the definition of UPFs themselves. The consensus is that UPFs are highly processed, energy-dense foods with plenty of bad ingredients (sugar, unhealthy fats, and salt) and few good ingredients (fiber, protein, vitamins, and minerals) that can be produced at low cost and have a long shelf life. The global reliance on these foods—in the United States of America and the United Kingdom, UPFs constitute more than half of the average individual’s total calorie consumption [2,3]—is considered a substantial contributor to overweight and obesity, thereby increasing risk of myriad adverse health effects [4]. While consumers may have a general sense of what UPFs are—ice cream, carbonated beverages, potato chips—most have little idea what’s really in them or why they are so bad. And many people would be surprised at the breadth of foods included under the UPF umbrella.

The most widely accepted food classification system used by nutrition researchers is the NOVA system—a system developed by a team of nutritionists to classify foods into 4 groups based on degrees of processing rather than ingredients and nutrients [5]. Despite the widespread use of NOVA, some researchers question its utility. Indeed, one study showed that food and nutrition specialists themselves struggled to accurately assign many foods to appropriate NOVA groups, with many of them deeming certain UPFs nutritionally acceptable [6]. The intent of NOVA is laudable, but if nutrition specialists struggle to use the system, how user-friendly will it be for the average consumer?

The definition of UPFs is not the only source of uncertainty. The field of nutritional epidemiology also involves many challenges, ranging from difficulties in assessing nutrition at the individual level—such as reliance on food diaries, which are prone to inaccuracies and recall bias—to applying appropriate statistical methods to understand complex associations between diet and health outcomes. For studies on UPFs specifically, it is often challenging to disentangle whether health effects are attributable to their high degree of processing, low nutritional quality, or to interrelated health factors (or a combination thereof) [7]. Indeed, good health involves much more than what we eat and drink; the influence of physical activity, limited screen time, and sleep (to name a few) is well studied, and these lifestyle factors are intimately intertwined on an individual level. It is equally difficult to contextualize the harms of UPFs and identify the degree of absolute risk they pose—a consideration nutritional studies often lack. These complexities are crucial to explore, particularly given that UPF consumption tends to be higher among individuals in the most deprived communities and low-resource settings [2,8], who already suffer from higher food insecurity and carry a disproportionate burden of poor health outcomes.

Against the backdrop of the challenges inherent to nutrition research, consumers are bombarded with conflicting messages—on the one side, direct health warnings from the news media that often oversimplify scientific evidence and exaggerate health risks, and on the other, aggressive marketing campaigns from the food industry promoting supposedly healthy eating habits. These marketing strategies are highly effective; sales of UPFs are growing steadily around the world, particularly in middle-income countries [9]. In the UK, one-third of food and drink advertising is dedicated to unhealthy options compared to only 1% for fruit and vegetables, according to a 2023 report from The Food Foundation charity [10]. The food industry also sponsors many scientific studies, which can contribute to mixed messages and threatens to undermine the credibility of nutrition science.

Policymakers in some countries, such as Brazil and Chile, are trying to level the playing field by introducing policies such as mandatory food and drink reformulations or tax levies on unhealthy items like sugar-sweetened beverages (SSBs). Some countries have also implemented marketing bans and front-of-package labelling for nonessential, “high-in” foods. Consumer advocacy groups in countries such as Mexico, South Africa, and Brazil have also played an influential role in supporting nutrition policies by mobilizing political support and challenging industry opposition. However, it is also critical to improve the accessibility and affordability of healthy, unprocessed foods to populations worldwide, and the time is overdue for governments and policymakers to prioritize these efforts. Initiatives such as the Supplemental Nutrition Assistance Program (SNAP) in the USA, for example, provide food assistance to families with low incomes to help promote healthy nutrition. Such projects can be challenging in view of lobbying work by food and drink companies; in the USA, US$106 million went to such lobbying in 2023 alone—nearly twice that of the tobacco and alcohol industries combined—according to an analysis by the Financial Times [11].

In support of efforts to improve population health, the United Nations (UN) has deemed 2016 to 2025 the Decade of Nutrition, in support of the Sustainable Development Goals, with an overriding mission to foster an environment that transcends income disparity, malnutrition challenges, and characteristics of food and health systems worldwide. The UN’s primary focus has been on the eradication of malnutrition—a goal that many governments are still failing to meet—but the crisis of overweight and obesity has now eclipsed that of malnutrition. According to WHO, in 2022, 2.5 billion adults and 390 million children and adolescents aged 5 to 19 years were overweight or obese, several times higher than those underweight [12].

The solutions to this ongoing nutrition crisis will inevitably be as complex as the problems, and all stakeholders must be held to higher standards. Nutrition research must strive for increased rigor. The food industry must go further to prove the nutritional quality and safety of new food products. The news media must curb their tendency toward sensationalism in favor of balanced, data-driven reporting. Governments and policymakers must push harder to promote industry transparency and address the relative lack of focus on healthy foods. Only an all-stakeholder, all-society approach will succeed in balancing the many competing forces that currently tip the scales toward UPFs and away from more healthy eating habits and food systems.

## Abbreviations

SNAP: Supplemental Nutrition Assistance Program
SSB: sugar-sweetened beverage
UN: United Nations
UPF: ultraprocessed food
